# Population pharmacokinetics of PF-05280014 (a trastuzumab biosimilar) and reference trastuzumab (Herceptin^®^) in patients with HER2-positive metastatic breast cancer

**DOI:** 10.1007/s00280-019-03850-1

**Published:** 2019-05-03

**Authors:** Xiaoying Chen, Cheryl Li, Reginald Ewesuedo, Donghua Yin

**Affiliations:** 1 Early Oncology Development and Clinical Research, Pfizer Inc., 10777 Science Center Drive, San Diego, CA 92121 USA; 2 Clinical Pharmacology/Pharmacometrics, Pfizer Essential Health Research and Development, Pfizer Inc, 300 Technology Square, Cambridge, MA 02140 USA; 3 Pfizer Essential Health, Biosimilars Clinical R&D, Pfizer Inc, 610 Main Street, Cambridge, MA 02139 USA

**Keywords:** Biosimilar, Pharmacokinetics, Trastuzumab, Human epidermal growth factor receptor-2-positive metastatic breast cancer, NONMEM, Population pharmacokinetics

## Abstract

**Purpose:**

PF-05280014 is a biosimilar to trastuzumab (Herceptin^®^). Following demonstration of pharmacokinetic (PK) similarity in healthy volunteers, a comparative clinical study in patients with HER2-positive metastatic breast cancer (mBC) compared the efficacy, safety and immunogenicity of PF-05280014 and trastuzumab sourced from the EU (trastuzumab-EU), both with paclitaxel.

**Methods:**

Population PK of PF-05280014 and trastuzumab-EU was evaluated.

**Results:**

Overall, 702 patients were treated: PF-05280014 (*n *= 349) and trastuzumab-EU (*n *= 353). Peak-and-trough serum drug concentration samples were collected (selected doses) following repeated intravenous administration of PF-05280014 or trastuzumab-EU. Population PK analysis was performed with drug concentration–time data to cycle 17 for each compound, using nonlinear mixed effect modeling. Potential baseline covariates (circulating HER2 concentrations, body weight, Japanese race, Eastern Cooperative Oncology Group status, number of metastatic sites and antidrug antibody status) were evaluated. Concentration–time data of PF-05280014 and trastuzumab-EU were adequately described by a two-compartment model with first-order elimination, with inter-individual variability (IIV) on clearance (CL), volumes of distribution in central compartment (*V*_1_) and peripheral compartments, and intercompartment clearance. Similar estimated PK parameters and IIV were obtained for both treatments. For PF-05280014 and trastuzumab-EU, baseline body weight was an influential covariate on CL and *V*_1_; the magnitude was comparable between treatments. PK was consistent between the limited number of Japanese and non-Japanese patients for both compounds.

**Conclusions:**

PF-05280014 and trastuzumab-EU had similar PK parameters and influential PK covariates in patients with HER2-positive mBC. These results provided further evidence in patients for PK similarity between PF-05280014 and trastuzumab-EU.

**Clinical trial registration:**

ClinicalTrials.gov, NCT01989676.

## Introduction

Trastuzumab (Herceptin^®^; Genentech, San Francisco, CA, USA) is a recombinant humanized IgG1 monoclonal antibody targeting human epidermal growth factor receptor-2 (HER2) [1, 2]. Biosimilars are biologic drugs that are highly similar to the corresponding approved originator (reference) biologic, with no clinically meaningful differences in quality, efficacy and safety between the two products [3].

PF-05280014 (Trazimera™) was recently approved by the European Medicines Agency as a biosimilar to trastuzumab sourced from the EU (trastuzumab-EU) for all eligible indications of the reference product in each region [4]. PF-05280014 has an identical amino acid sequence, with similar physicochemical and functional properties as trastuzumab sourced in the United States (trastuzumab-US) and trastuzumab-EU in nonclinical assessments [5].

A phase 1 study in healthy male subjects demonstrated that the pharmacokinetics (PK) of PF-05280014 were similar to both trastuzumab-US and trastuzumab-EU [6]. In this study, healthy subjects were randomized to receive a single 6 mg/kg intravenous (IV) dose of PF-05280014, trastuzumab-US or trastuzumab-EU. PK parameters were estimated using a noncompartmental approach. The 90% confidence intervals (CIs) for the ratios of exposure parameters, including maximum serum concentration (*C*_max_), area under the serum concentration–time profile (AUC) from time zero to the last time point with quantifiable concentration (AUC_tlast_) and extrapolated to infinite time (AUC_0–∞_), were within 80–125% for the pairwise comparisons among the three treatment groups.

In a comparative clinical study in patients with HER2-positive metastatic breast cancer (mBC), the efficacy, safety and immunogenicity of PF-05280014 demonstrated similar objective response rate, safety and immunogenicity to trastuzumab-EU (both in combination with paclitaxel) [7]. Moreover, peak-and-trough drug concentrations were collected for selected doses to support the assessment of population PK of PF-05280014 and trastuzumab-EU. While PK similarity was demonstrated in the phase 1 study in healthy subjects [6], it is expected that PK assessment in this comparative clinical study could provide additional evidence of PK similarity of the compounds in a population of patients with breast cancer, and allow the evaluation of the effects of covariate factors on PK of PF-05280014 and trastuzumab-EU. This manuscript reports the population PK modeling analysis based on data from the comparative clinical study in patients with HER2-positive mBC.

## Methods

### Study population and design

A total of 707 patients were enrolled in this multinational, double-blind, randomized, comparative clinical trial evaluating the efficacy, safety, PK and immunogenicity of PF-05280014 versus trastuzumab-EU (both in combination with paclitaxel) in patients with HER2-positive mBC in the first-line treatment setting [7]. This study was registered at ClinicalTrials.gov (NCT01989676).

In total, 702 of the randomized patients received study treatment. Of these, 349 and 353 patients were enrolled in the PF-05280014 and trastuzumab-EU treatment groups, respectively. Details of the study drug treatments have been described previously [7]. Briefly, PF-05280014 or trastuzumab-EU was administered weekly (first dose of 4 mg/kg, IV infusion administered over 90 min, with subsequent doses of 2 mg/kg, infused over 30–90 min, depending on tolerability) on days 1, 8, 15 and 22 of each 28-day cycle, and until at least week 33. Following completion of the paclitaxel administration period, and starting no earlier than week 33, the PF-05280014 or trastuzumab-EU regimen could be changed to 6 mg/kg, infused over 30–90 min every 3 weeks, at the discretion of the investigator. Paclitaxel was administered on days 1, 8 and 15 of each 28-day cycle (starting dose of 80 mg/m^2^, IV infused over 60 min). In the absence of disease progression or unacceptable toxicity in the judgement of the investigator, paclitaxel treatment was continued for at least six cycles or until maximal benefit of response was obtained.

### Pharmacokinetic evaluations

Population PK assessment of PF-05280014 and trastuzumab-EU was conducted using all serum drug concentration–time data up to and inclusive of cycle 17, day 1 as of the primary completion date (PCD) cutoff of August 24, 2016 (data snapshot date of October 12, 2016), except the end-of-treatment (EOT) visit and unplanned records. The PCD refers to the data cut for the primary efficacy analysis when all patients had either completed the week 33 tumor assessment or discontinued study drug earlier than the week 33 visit. For cycles 1–8, pre-dose drug concentration samples (taken within 4 h before the start of infusion) were collected on day 1 of cycles 1, 3, 4, 5, 7 and 8, and additionally on day 8 of cycles 1 and 5. In addition, end of infusion drug concentration samples were collected 1 h after the end of infusion on day 1 of cycles 1 and 5. For subsequent 28-day cycles, drug concentration samples were collected every 3 cycles on day 1 before infusion, and at any time during the end-of-treatment visit.

Drug concentration samples were analyzed by QPS LLC (Newark, DE, USA) using a validated enzyme-linked immunosorbent assay (ELISA) [6]. Drug concentrations were determined by interpolation from calibration standard curves in the range of 0.5–100 mg/L.

Serum samples for the assessment of antidrug antibodies (ADAs) and neutralizing antibodies (NAbs) were collected at pre-dose of cycle 1, 3, 5, 8 and every 3 cycles until the end-of-treatment visit. Two electrochemiluminescent immunoassays, one for detecting antibodies against PF-05280014 and the other for detecting antibodies against trastuzumab-EU, were used to analyze the samples. Both ADA assays utilized the same immunoassay platform and were validated in accordance with regulatory guidance documents [6]. ADA sample analyses were conducted by Intertek Pharmaceutical Services (San Diego, CA, USA), and followed a tiered approach of screening, confirmation and titer determination [6]. If a sample tested positive for ADA against the dosed product, the sample was also tested for cross-reactivity against the other product, using the corresponding assay. Samples that tested positive for the presence of ADAs (anti-PF-05280014 or anti-trastuzumab-EU) were further analyzed for NAbs using validated competitive binding assays, at QPS LLC.

In addition, serum samples were collected at pre-dose of cycles 1, 3, 5, 8 and at the end-of-treatment visit, and analyzed by Q2 Solutions (Morrisville, NC, USA) for soluble, shed HER2 extracellular domain concentrations using a HER2 ELISA development kit (Nuclea Diagnostics, Pittsfield, MA, USA).

### Population PK analysis

#### General modeling approach

Population PK assessment was conducted using a nonlinear mixed effect modeling (NONMEM) approach. The proposed models were implemented in NONMEM (ICON Development Solutions, Ellicott City, MD, USA) version 7.2 for model parameter estimations using appropriate estimation methods (e.g., first-order conditional estimation method as implemented in NONMEM (FOCE)]. Perl-speaks-NONMEM (PsN) version 4.2.0 was used for stepwise covariate modeling (SCM), visual predictive check (VPC) and nonparametric bootstrapping; R (version 3.2.2) was used for pre- and post-processing and for graphical presentation of the results.

The analysis followed the stepwise approach of base model development, covariate model development, final model development and adequacy assessment (e.g., goodness of fit and VPC). A population PK model was developed for both treatment groups (PF-05280014 and trastuzumab-EU) separately, to allow for comparison of the model structures and parameter estimates between the two treatment groups. All patients who had at least one drug concentration sample collected post-dose during the period up to cycle 17, day 1 were included in the analysis. Since the number of post-dose samples with concentration less than the lower limit of quantification (LLOQ) was low (43 below the limit of quantification values of 7098 total observations) in this study, the M1 method was implemented for the population PK modeling, in which post-dose samples with concentrations < LLOQ were excluded.

#### Structural PK model and variability models

Based on the literature [8], a two-compartment model was used as the starting structural model. Since the range of observed concentrations was wide (> 2 orders of magnitude), the concentration data were log-transformed before model fitting. The inter-patient variability in PK parameters, including clearance (CL), intercompartmental clearance (*Q*), volumes of distribution in the central compartment (*V*_1_) and peripheral compartment (*V*_2_), were assumed to be log-normally distributed (exponential model). For the variance–covariance matrix (*Ω*) of inter-individual random effects, the diagonal matrix was applied as there were no significant correlations between CL and *V*_1_ during model development. The residual error was described using an additive error model, after log-transformation of the PK data.

#### Covariate evaluations

Based on the literature of the population PK analysis of trastuzumab [8] and key characteristics of the patients enrolled in the study, potential covariates evaluated for both CL and *V*_1_ included baseline HER2 concentrations, selected demographics (e.g., body weight, Japanese ethnicity and race), selected patient characteristics [Eastern Cooperative Oncology Group (ECOG) status and number of metastatic sites] and ADA status (Table 1).

**Table 1:** Covariates included in the population PK analysis

PK parameter	Covariate
CL	BWT, HER2STAT, JAPA, RACE_STAT, ADA_BL, ECOG and N_META
*V*_1_	BWT, HER2STAT, JAPA, RACE_STAT, ADA_BL, ECOG and N_META

*ADA* antidrug antibody, *ADA_BL* baseline ADA status, *BWT* baseline body weight, *CL* linear clearance, *ECOG* baseline Eastern Cooperative Oncology Group status, *HER2STAT* baseline human epidermal growth factor receptor-2 (HER2) concentration, *JAPA* Japanese vs. non-Japanese, *N_META* number of metastatic sites, *PK* pharmacokinetics, *RACE_STAT* Asian vs. non-Asian, *V*_*1*_ volume of distribution in central compartment

Continuous variables were tested using a power model, following the equation:

1 $${\text{TVP}}_{j} = P_{\text{pop}} \times \left( {\frac{{{\text{COV}}_{j} }}{{{\text{COV}}_{\text{median}} }}} \right)^{\theta},$$

where TVP_j_ represents the model-predicted PK parameter for the typical *j*th individual with normalized covariate value (COV_*j*_/COV_median_), *P*_pop_ represents the population central tendency for the PK parameter at the median covariate value, and *θ* represents the estimated scale factor.

Categorical covariates (e.g., Japanese ethnicity or number of metastatic sites) were modeled as a fractional change using the general equation:

2 $${\text{TVP}}_{j} = P_{\text{pop}} \times (1 + \theta),$$

where for the *X* groups within a given category,

- if COV = group 1, *θ* = 0,
- if COV = group 2, *θ* = θ_1_,
- …
- if COV = group *X*, *θ* = *θ*_*X*−1_.

Stepwise covariate modeling was used to evaluate the covariates. Inclusion of covariates in the full PK model was based on the likelihood ratio test (LRT) to compare nested models, and implemented in a forward inclusion stepwise procedure. A significance value of *α* = 0.05 was implemented during the forward selection process to assess the significance of including covariate(s) in a stepwise manner, to establish the full model. Final model development began with the full model, with a backward elimination algorithm, using LRT at a significance level of *α* = 0.001.

### Model goodness of fit and evaluation

Goodness of fit of models was evaluated during model development using criteria including change of objective function value, condition number, visual inspection of different diagnostic plots, precision of the parameter estimates, and decreases in inter-individual variability and residual variability.

A bootstrap resampling technique was used to evaluate the stability of the final model [9] and to estimate CIs of the parameter estimates. The final model was fitted to 1000 replicate datasets, which were generated by randomly sampling the original data with replacement. The median and 95% CI of the estimated parameters from the replicate datasets were calculated and compared with the point estimates from the original dataset.

### Model predictive performance and assessments of simulated concentrations between treatments or patients

Model predictive performance was evaluated using VPC, by simulation of new observations for 1000 trials using the final model parameter estimates (fixed and random effects) and observed data structure (dosing records, observation times, covariate values, etc.). The median and quantiles of the observed data were compared with those of the simulated data. The simulated PK concentrations were subsequently compared between treatment groups. Model-simulated concentrations from the 1000 trial simulations were presented as box plots for PF-05280014 and trastuzumab-EU, to compare the predicted concentrations in the two treatment groups. Another comparison was conducted for drug concentrations between Japanese patients and all patients. The observed drug concentrations for Japanese patients were plotted together with the model-simulated drug concentrations for all patients.

## Results

### Determination of the structural PK model

Based on the published literature of population PK of trastuzumab [8], a two-compartment model with first-order elimination and zero-order input (constant rate infusion) was chosen as the starting structural model. Inter-individual variability in the parameters of CL, *V*_1_, *V*_2_ and *Q* was assumed to be log-normally distributed (exponential model). With the same model structures, the base models for both treatment groups achieved satisfactory precision for parameter estimates, and the diagnostic plots indicated overall reasonable model fitting (data not shown).

### Covariate effects on PK parameters

Potential covariates evaluated for CL and *V*_1_, including baseline circulating HER2 concentrations, body weight, Japanese ethnicity, race, ECOG status, number of metastatic sites and ADA status at baseline, are shown in Table 1.

The final models identified identical covariates for PF-05280014 and trastuzumab-EU, with baseline body weight as a covariate affecting CL and *V*_1_. In addition, the magnitude of the covariate effect was similar between treatment groups. Body weight had a similar impact on CL (with a power factor of 0.637 for PF-05280014 and 0.673 for trastuzumab-EU) and on *V*_1_ (with a power factor of 0.507 for PF-05280014 and 0.512 for trastuzumab-EU).

### The final model

Overall, the PK parameter estimates in the final models were similar between PF-05280014 and trastuzumab-EU (Table 2). The estimated CL values of PF-05280014 and trastuzumab-EU were 0.0104 L/h and 0.00948 L/h, respectively. The estimated central compartment volume (*V*_1_) was 3.15 L and 3.10 L for PF-05280014 and trastuzumab-EU, respectively. The estimated *Q* values were 0.0194 L/h and 0.0186 L/h, and *V*_2_ values were 5.55 L and 5.66 L for PF-05280014 and trastuzumab-EU, respectively.

**Table 2:** Parameter estimates and confidence intervals from the final models

Parameter	PF-05280014		Trastuzumab-EU	
	NONMEM results	Nonparametric bootstrap	NONMEM results	Nonparametric bootstrap
	Estimate (95% CI)^a^	Estimate (median) (95% CI)^b^	Estimate (95% CI)^a^	Estimate (median) (95% CI)^c^
*V*_1_ (L)	3.15 (2.99–3.31)	3.15 (3.04–3.27)	3.10 (2.91–3.29)	3.10 (2.94–3.27)
*V*_2_ (L)	5.55 (5.24–5.86)	5.59 (4.80–6.59)	5.66 (5.12–6.20)	5.65 (4.70–6.71)
CL (L/h)	0.0104 (0.0098–0.0110)	0.0103 (0.0099–0.0109)	0.00948 (0.009–0.010)	0.00948 (0.0091–0.0099)
*Q* (L/h)	0.0194 (0.0160–0.0228)	0.0192 (0.0167–0.0224)	0.0186 (0.017–0.020)	0.0186 (0.0164–0.0228)
BWT effect on *V*_1_	0.507 (0.316–0.698)	0.513 (0.348–0.666)	0.512 (0.026–0.998)	0.518 (0.295–0.725)
BWT effect on CL	0.637 (0.450–0.824)	0.638 (0.474–0.801)	0.673 (0.430–0.916)	0.672 (0.494–0.838)
*V*_1_ ω^2^ (%CV)	0.0405 (20) (0.011–0.070)	0.040 (0.013–0.076)	0.123 (35) (0.061–0.185)	0.122 (0.064–0.189)
*V*_2_ ω^2^ (%CV)	1.06 (103) (0.770–1.350)	1.057 (0.730–1.361)	1.08 (104) (0.772–1.388)	1.056 (0.743–1.407)
CL ω^2^ (%CV)	0.0934 (31) (0.072–0.115)	0.091 (0.070–0.121)	0.0687 (26) (0.054–0.084)	0.067 (0.053–0.096)
*Q* ω^2^ (%CV)	0.504 (71) (0.332–0.676)	0.491 (0.296–0.704)	0.528 (73) (0.339–0.717)	0.519 (0.336–0.762)
Res Add Err	0.272 (0.243–0.301)	0.271 (0.245–0.301)	0.292 (0.249–0.335)	0.292 (0.255–0.331)

*BWT* baseline body weight, *CI* confidence interval, *CL* systemic clearance, *%CV* percent coefficient of variation, *NONMEM* nonlinear mixed effect modeling, *Q* intercompartment clearance, *Res Add Err* residual additive error, *SE* standard error, *trastuzumab*-*EU* trastuzumab sourced from the European Union, *V*_*1*_ volume of distribution in central compartment, *V*_*2*_ volume of distribution in peripheral compartment

^a^The 95% CI was manually calculated using equation: estimate ± 1.96 × SE. Standard error was obtained from the covariance step using the *R*/*S* matrix in NONMEM

^b^The bootstrap runs which had successful minimization (916 out of 1000) were included in the calculation of the 95% CI. The 95% CI represent 2.5th to 97.5th percentiles of the included bootstrap estimates

^c^The bootstrap runs which had successful minimization (932 out of 1000) were included in the calculation of the 95% CI. The 95% CI represent 2.5th to 97.5th percentiles of the included bootstrap estimates

**Fig. 1:**
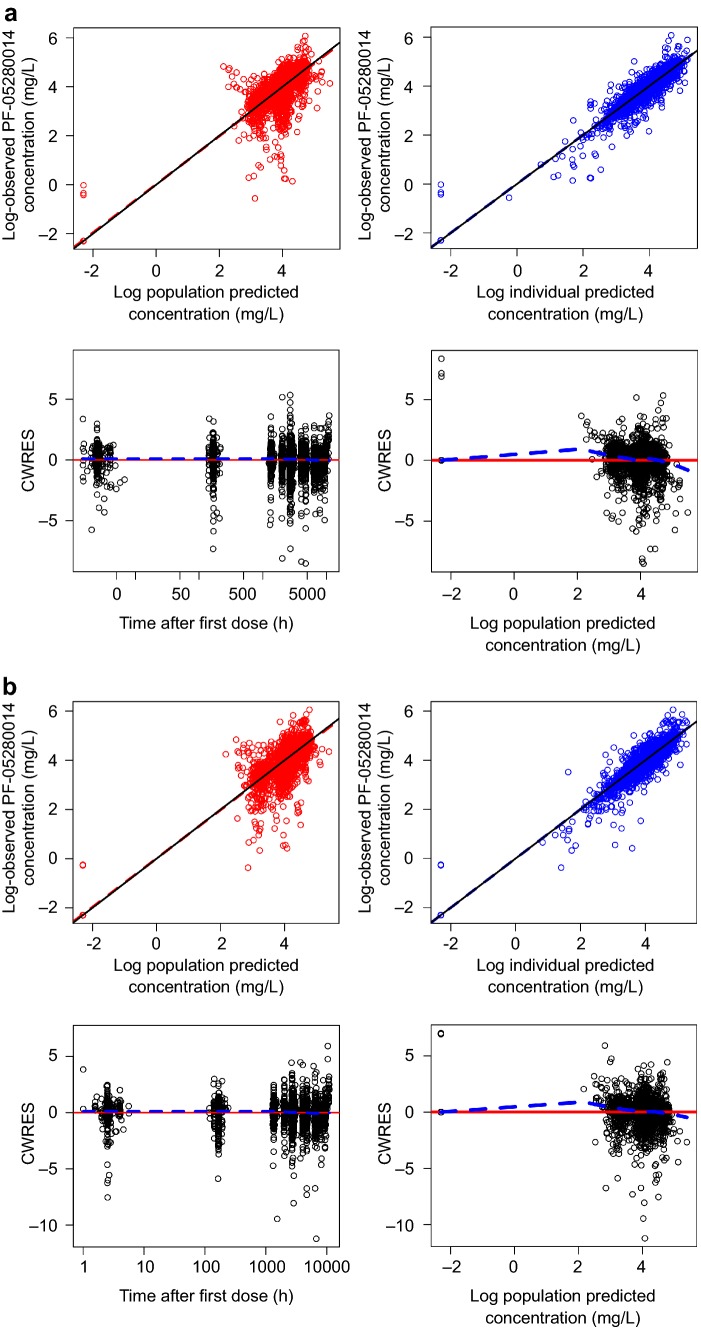
Diagnostic plots for final models of **a** PF-05280014 and **b** trastuzumab-EU groups. *CWRES* conditional weighted residuals, *LOWESS* locally weighted scatterplot smoothing trend line, *trastuzumab*-*EU* trastuzumab sourced from the European Union. In the scatter plots of observations versus predictions, the solid line and dashed line show the reference line (diagonal line) and linear regression line based on the individual data points, respectively. In the scatter plots of residuals, the solid line and dashed line show reference line (*y* = 0) and LOWESS, respectively. Observed concentrations and individual predictions were log-transformed

Final model parameter and *η*/*ε*-shrinkage values were estimated with acceptable precision. The diagnostic plots for the final models of PF-05280014 and trastuzumab-EU are presented in Fig. 1. Overall, the population prediction tracked well with the central tendency of the observed data. Conditional population weighted residuals (CWRES) plotted against population-predicted values (PRED) and time after the first dose did not indicate any appreciable model misspecification of the structural model or the residual error model. The *η* estimates appeared normally distributed and displayed medians near zero for CL, *V*_1_, *V*_2_ and *Q* (results not shown).

A bootstrap resampling technique was used to evaluate the stability of the final model and to estimate CIs of the parameter estimates. Median parameter estimates and the 95% CIs from the bootstrap analyses were in good agreement with the point estimates from the final model, indicating reasonable stability of the final model (Table 2). The significance of covariates included was further supported by the bootstrap analyses, as none of the 95% CIs for the covariate effects included zero. Importantly, the parameter estimates and their 95% CIs generated from the bootstrap analyses were similar between the treatment groups, and were similar to the observations from the final model.

### Assessment of model predictive performance

**Fig. 2:**
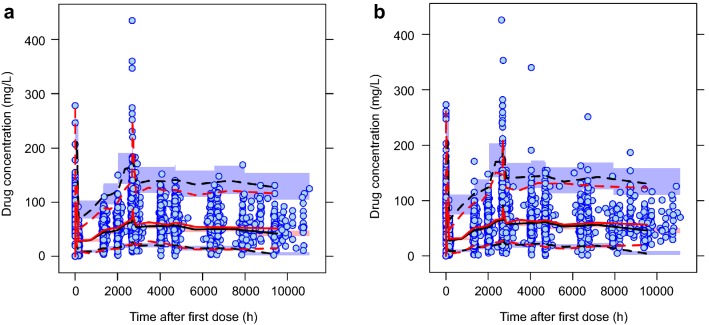
Visual predictive check for the final **a** PF-05280014 and **b** trastuzumab-EU models. *Trastuzumab*-*EU* trastuzumab sourced from the European Union. Blue circles represent the observed data and the red lines represent the median (solid line), 2.5th percentile (dashed line), and 97.5th percentile (dashed line) of the observed data. For 1000 simulated trials, the median, 2.5th percentile and 97.5th percentile of simulated concentrations were calculated for each time bin and are presented by black lines. The 95% confidence intervals for the simulated median and each percentile are shown by light pink and light blue shaded areas

The final model was evaluated using a VPC approach. Separate plots with time ranges based on the actual sampling time of observed data after the first dose for PF-05280014 and trastuzumab-EU are shown in Fig. 2. Overall, the model simulation reproduced the observed longitudinal concentration profiles successfully. Importantly, the simulated concentration–time profiles were similar between the two treatment groups.

### Comparison of model-simulated PK for PF-05280014 and trastuzumab-EU

**Fig. 3:**
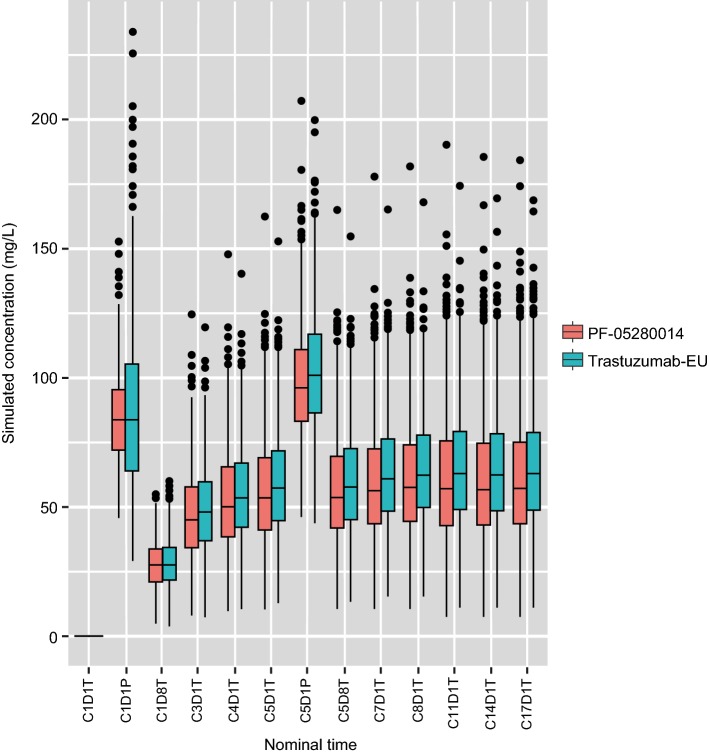
Simulated concentrations for PF-05280014 and trastuzumab-EU using the final models. *Trastuzumab*-*EU* trastuzumab sourced from the European Union. The nominal time points for simulation were plotted on the *x*-axis. The notation “C*x*D*y*T” stands for “Cycle *x* Day *y* Trough” and “C*x*D*y*P” stands for “Cycle *x* Day *y* Peak”. The trough concentration is the simulated pre-dose concentration; the peak concentration is the simulated concentration at 1 h after the end of infusion

The VPC simulations based on the final models indicated that model-predicted concentrations were similar between the PF-05280014 and trastuzumab-EU treatment groups (Fig. 3). In addition, model-predicted PK parameters were similar between the two treatment groups (Table 2), and were generally consistent with published PK parameters for Herceptin^®^ [1].

### Comparison of observed PK in Japanese patients to simulated concentrations in all patients

**Fig. 4:**
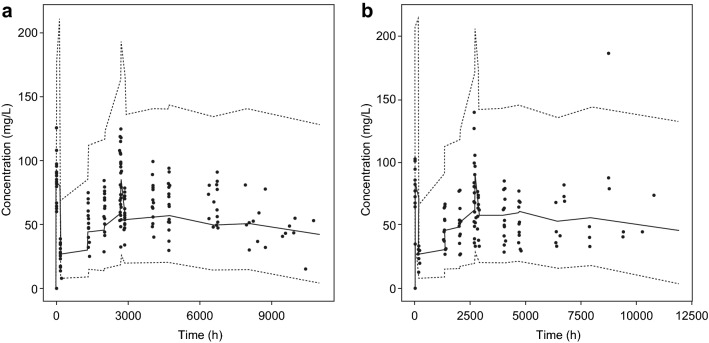
Observed concentrations in Japanese patients vs. visual predictive check in all patients, stratified by **a** PF-05280014 and **b** trastuzumab-EU treatment groups. *Trastuzumab*-*EU* trastuzumab sourced from the European Union. The black dots represent the observed concentration in Japanese patients. The black lines represent the median (solid line), 2.5th percentile (dashed line) and 97.5th percentile (dashed line) of the simulated concentrations from 1000 simulated trials

As the number of Japanese patients was limited (*n* = 18 for PF-05280014 and *n* = 14 for trastuzumab-EU), the observed concentrations for individual Japanese patients were plotted together with the VPC simulation for all patients, stratified by treatment groups (Fig. 4). The observed concentrations in Japanese patients were within the 95% CIs of the VPC simulations for all patients, suggesting that the PK of Japanese patients was similar to that of all patients in both treatment groups.

## Discussion

Biosimilars are a growing class of biotherapeutics that could provide alternatives to the corresponding originator biologics, with similar efficacy and safety, while offering increased patient access to these important medicines [10, 11]. Owing to the molecular structure and the production process for biotherapeutics, biosimilars are not identical to their originator biologics, and thus follow a totality-of-the-evidence approach for demonstrating biosimilarity. As well as preclinical assessments such as physicochemical, biologic and preclinical studies to establish biosimilarity, the aim of clinical development is to confirm the safety and efficacy, in addition to PK and pharmacodynamics, in comparison with the originator (reference) biologic [3, 12, 13].

Clinical pharmacology assessment is one of the pillars constituting the totality of evidence for biosimilarity. In general, clinical pharmacology assessments for biosimilars mainly focus on PK and immunogenicity. Typically, PK similarity is first demonstrated in a comparative clinical study in healthy volunteers, where possible, using a noncompartmental analysis (NCA) approach, in which similar exposure for the potential biosimilar and the originator biologic may support the demonstration of biosimilarity [3]. Although comparative clinical studies mainly evaluate similarity in efficacy, safety and immunogenicity, PK evaluation can provide further supportive evidence for biosimilarity, particularly in relevant patient populations with the intended dose and dosing regimen.

This manuscript presents a case study for applying population PK modeling to a comparative clinical study, further supporting biosimilarity. Owing to the limitations of the sparse PK sampling in comparative clinical studies, PK similarity assessment using NCA is not feasible. Rather, population PK modeling could be applied to evaluate the PK parameters, inter-subject variabilities in PK, and significant covariates that impact PK parameters in comparative clinical studies, where the patient population and dosing regimens are most relevant.

This population PK analysis characterized the PK of PF-05280014 and trastuzumab-EU, and evaluated the effect of different potential covariates on the PK of these two treatment groups in patients with HER2-positive mBC. For both treatment groups, the final PK models had identical two-compartmental model structures with inter-patient variability on CL, *V*_1_, *V*_2_ and *Q*, and baseline body weight as an influential covariate on both CL and *V*_1_.

This population PK modeling showed similar estimates for PK parameters between PF-05280014 and trastuzumab-EU in patients with HER2-positive mBC. The CL values of PF-05280014 and trastuzumab-EU were similar between treatment groups, and were within the reported range for CL in the literature using population PK modeling [1, 2, 8]. In addition, other structure PK parameters including *V*_1_ and *Q* values were similar between the two treatments and were also within the reported range of values [8]. The *V*_2_ estimates, while slightly higher than the reported value of 4.8 L [8], were similar between treatment groups (PF-05280014: 5.55 L and trastuzumab-EU: 5.66 L). Moreover, the inter-individual variability was generally consistent between the two treatment groups.

For both treatment groups, baseline body weight was identified as the covariate influencing CL and *V*_1_. The final model indicated that body weight is an important determinant of drug exposure. Body weight influenced CL (with a power factor of 0.637 for PF-05280014 and 0.673 for trastuzumab-EU) and *V*_1_ (with a power factor of 0.507 for PF-05280014 and 0.512 for trastuzumab-EU). Body weight has been previously reported as a covariate on *V*_1_, with a power factor of 0.556 [8]. Other potential covariates reported in the literature [8], such as the number of metastatic sites on CL and baseline HER2 concentrations on CL and *V*_1_, were not identified to be significant covariates in the final models in this analysis. One possible explanation is that the significance level for selecting the covariates in the current analysis is more stringent (*p* < 0.001) than reported in the literature (*p* < 0.005) [8].

A bootstrap technique was applied to further test the model stability. Parameter estimates from the bootstrap technique were highly similar to the point estimate values from the final model run, suggesting that the final models were stable and provided reliable post hoc estimates of the PK parameters. More importantly, the bootstrap estimates were also very similar between the two treatment groups.

Based on the final models, a VPC method was employed to simulate concentration–time profiles for PF-05280014 and trastuzumab-EU. Consistency between the observed PK data and the model-simulated PK data further supported adequacy of the final models, demonstrating that the final models captured the distribution of the observed data appropriately. The simulated PK data for the two treatment groups largely overlapped over the course of the assessment period, providing evidence that the PK of the two products were similar in this patient population.

The potential use of a population PK approach to support the extrapolation of PK similarity to selected ethnic groups was also explored. For biosimilar development, it may not be efficient to repeat the PK similarity study in different ethnic groups to support regional registrations. As such, a population PK approach could explore whether ethnicity is a covariate influencing PK using data from a global study in a sufficient number of patients from a particular ethnic group. This could provide evidence of similar PK between a particular ethnic group and the global population for the biosimilar and originator biologic, which in turn could support the extrapolation of PK similarity results to ethnic groups. In the current analysis, covariates related to ethnicity—including Japanese ethnicity and race (Asian vs. non-Asian)—were evaluated but were not identified to be significant covariates in the final models, suggesting neither Japanese ethnicity nor Asian race substantially impact drug exposure. In addition, the observed exposure in Japanese patients (less than 5% of the enrolled patients) was compared with simulated exposure in all patients, based on the final models, suggesting the drug exposures in Japanese patients was similar to the overall population. In summary, the population PK analysis did not reveal any appreciable differences in PK between the Japanese and non-Japanese patients in either the PF-05280014 or trastuzumab-EU treatment groups. As PK similarity has been demonstrated in non-Japanese patients, the evidence of similar PK between Japanese and non-Japanese patients for both PF-05280014 and trastuzumab-EU could offer supportive information regarding PK similarity of PF-05280014 or trastuzumab-EU in the Japanese population.

In conclusion, this population PK analysis further confirmed that the PK of PF-05280014 and trastuzumab-EU are similar in patients with HER2-positive mBC. Previously, PK similarity was demonstrated in a study in healthy subjects [6]. In a comparative clinical study in patients with operable HER2-positive breast cancer receiving neoadjuvant chemotherapy, PF-05280014 demonstrated non-inferior PK and similar efficacy, safety and immunogenicity to trastuzumab-EU [14].

This analysis also showed that in the limited number of Japanese patients, the PK appeared to be largely consistent with the non-Japanese population for both treatment groups. In conjunction with the previous demonstration of PK similarity between PF-05280014 and trastuzumab-EU in a study in healthy subjects conducted in the United States [6], these results from the population PK analysis suggest that the PK of PF-05280014 and trastuzumab-EU are likely to be similar in Japanese patients.

## Data Availability

Upon request, and subject to certain criteria, conditions and exceptions (see https://www.pfizer.com/science/clinical-trials/trial-data-and-results for more information), Pfizer will provide access to individual de-identified participant data from Pfizer-sponsored global interventional clinical studies conducted for medicines, vaccines and medical devices (1) for indications that have been approved in the US and/or EU, or (2) in programs that have been terminated (i.e., development for all indications has been discontinued). Pfizer will also consider requests for the protocol, data dictionary, and statistical analysis plan. Data may be requested from Pfizer trials 24 months after study completion. The de-identified participant data will be made available to researchers whose proposals meet the research criteria and other conditions, and for which an exception does not apply, via a secure portal. To gain access, data requestors must enter into a data access agreement with Pfizer.
