# Correction to: Population pharmacokinetics of PF‑05280014 (a trastuzumab biosimilar) and reference trastuzumab (Herceptin^®^) in patients with HER2‑positive metastatic breast cancer

**DOI:** 10.1007/s00280-019-03890-7

**Published:** 2019-07-10

**Authors:** Xiaoying Chen, Cheryl Li, Reginald Ewesuedo, Donghua Yin

**Affiliations:** 1 Early Oncology Development and Clinical Research, Pfizer Inc., 10777 Science Center Drive, San Diego, CA 2121 USA; 2 Clinical Pharmacology/Pharmacometrics, Pfizer Essential Health Research and Development, Pfizer Inc, 300 Technology Square, Cambridge, MA 02140, USA; 3 Pfizer Essential Health, Biosimilars Clinical R&D, Pfizer Inc, 610 Main Street, Cambridge, MA 02139 USA

## Correction to: Cancer Chemotherapy and Pharmacology (2019) 84:83–92 10.1007/s00280-019-03850-1

The article “Population pharmacokinetics of PF-05280014 (a trastuzumab biosimilar) and reference trastuzumab (Herceptin^®^) in patients with HER2-positive metastatic breast cancer” written by Xiaoying Chen, Cheryl Li, Reginald Ewesuedo, Donghua Yin was originally published electronically on the publisher’s internet portal (currently SpringerLink) on 3rd May 2019 without open access.

With the author(s)’ decision to opt for Open Choice the copyright of the article changed on 15th June 2019 to © The Author(s) 2019 and the article is forthwith distributed under the terms of the Creative Commons Attribution 4.0 International License (http://creativecommons.org/licenses/by/4.0/), which permits use, duplication, adaptation, distribution and reproduction in any medium or format, as long as you give appropriate credit to the original author(s) and the source, provide a link to the Creative Commons license and indicate if changes were made. The original article has been corrected.

